# Phylogeography of higher Diptera in glacial and postglacial grasslands in western North America

**DOI:** 10.1186/s12898-019-0266-4

**Published:** 2019-12-20

**Authors:** Anna M. Solecki, Jeffrey H. Skevington, Christopher M. Buddle, Terry A. Wheeler

**Affiliations:** 1 Department of Integrative Biology, University of Guelph, Guelph, ON N1G 2W1 Canada; 2 Natural Resource Sciences, McGill University, Macdonald Campus, Ste-Anne-De-Bellevue, QC H9X 3V9 Canada; 3 Agriculture and Agri-Food Canada, Canadian National Collection of Insects, Arachnids, and Nematodes, 960 Carling Ave, Ottawa, ON K1A 0C6 Canada

**Keywords:** Beringia, Chloropidae, COI, Cyt *b*, Heleomyzidae, Holocene, Nearctic, Pleistocene, Refugium

## Abstract

**Background:**

Pleistocene glaciations have had an important impact on the species distribution and community composition of the North American biota. Species survived these glacial cycles south of the ice sheets and/or in other refugia, such as Beringia. In this study, we assessed, using mitochondrial DNA from three Diptera species, whether flies currently found in Beringian grasslands (1) survived glaciation as disjunct populations in Beringia and in the southern refugium; (2) dispersed northward postglacially from the southern refugium; or (3) arose by a combination of the two. Samples were collected in grasslands in western Canada: Prairies in Alberta and Manitoba; the Peace River region (Alberta); and the southern Yukon Territory. We sequenced two gene regions (658 bp of cytochrome *c* oxidase subunit I, 510 bp of cytochrome *b*) from three species of higher Diptera: one with a continuous distribution across grassland regions, and two with disjunct populations between the regions. We used a Bayesian approach to determine population groupings without a priori assumptions and performed analysis of molecular variance (AMOVA) and exact tests of population differentiation (ETPD) to examine their validity. Molecular dating was used to establish divergence times.

**Results:**

Two geographically structured populations were found for all species: a southern Prairie and Peace River population, and a Yukon population. Although AMOVA did not show significant differentiation between populations, ETPD did. Divergence time between Yukon and southern populations predated the Holocene for two species; the species with an ambiguous divergence time had high haplotype diversity, which could suggest survival in a Beringian refugium.

**Conclusions:**

Populations of Diptera in Yukon grasslands could have persisted in steppe habitats in Beringia through Pleistocene glaciations. Current populations in the region appear to be a mix of Beringian relict populations and, to a lesser extent, postglacial dispersal northward from southern prairie grasslands.

## Background

Pleistocene glaciations have left their mark on North America, affecting topography and drainage systems, as well as biota. Changes in climate altered species distributions and community compositions, forcing species southward or into other refugia [1].

Although the ice receded approximately 10,000 years ago, the effects of the last glaciation are still felt: the continent is still rebounding [2], many species are left in remnants of their former range where a suitable microclimate has persisted [3] and populations, once disconnected by ice, still bear the genetic signature of former separation [4, 5]. However, these patterns are intricate due to the complicated nature of landscape changes during glacial cycles; Milankovitch cycles [6] shaped glacial movements during the Pleistocene, creating cycles of glacial states and interglacials, and asynchronous oscillations of regional ice margins [2].

Most species survived past glaciations south of the ice sheets [7, 8]. However, the large landmass of Beringia remained unglaciated in the northwest, and was a refugium or a series of refugia during the Pleistocene. The body of evidence that supports the Beringia refugium hypothesis spans many fields such as geology, palynology, biogeography, phylogeography and paleontology [9, 10].

The biogeographic patterns of many Beringian taxa have been explored to infer the glacial history of the region (e.g., [4, 5]). However, because some species assemblages from glaciated Beringia have no modern analogue [10–13], untangling the history of the Beringian biota is complex.

Xeric grasslands in the Yukon Territory and Alaska are rare and distinctive environments. Characterised by prairie sage (*Artemisia frigida* Willd.), bunch-grasses and forbs, these isolated communities are associated with arid, exposed, south-facing slopes and have a unique insect fauna. Although located mostly in the southern Yukon, there are northern outliers on steep slopes near Old Crow and along the Yukon and Firth Rivers [14].

To date, paleoecological and distributional data have been used to infer the origin of these grasslands (e.g., [11, 15, 16]). Fossil evidence suggests that these communities are analogues of the late Pleistocene arctic steppe ecosystem [11–13, 17]. Regional aridity during the Wisconsinan glaciation would have allowed this xeric steppe-like flora to be widespread [12, 18]. Subsequent climatic changes have reduced this ecosystem to a few arid, exposed areas.

Some plant and insect distributions suggest that some species in these grasslands may have a southern origin [15, 16] when warm, dry conditions during the Holocene Hypsithermal warm period could have allowed northward expansion of grassland biota. Subsequent cooling, and recession of grasslands, would have left disjunct populations in sites with warmer, drier microclimates. This has been suggested for the occurrence of disjunct northwestern grasslands in the Peace River region of Alberta, up to 54° N [19, 20], but has not been formally tested in the context of xeric Yukon grasslands.

Solecki et al. [21] analysed the community structure of the family Chloropidae (Diptera) in Yukon xeric grasslands, and in two grassland regions farther south (disjunct grasslands in the Peace River region, Alberta, and in the main body of the prairies in southern Alberta to Manitoba), and found that the Yukon assemblages are distinct from those in southern prairies, and suggested that the Yukon assemblages were a mix of species that persisted in Beringia through glaciation and species that dispersed northward postglacially. Some species in that study were present in all grassland regions studied, but even those widely distributed species may retain, at the population level, genetic evidence of isolation into Beringian and southern populations during glaciation. Such widespread species are the focus of this study.

The objective of this study was to (1) obtain molecular data on population structure of flies in western grasslands (including southern prairie grasslands, Peace River grasslands and Yukon grasslands) and (2) evaluate these population patterns in the context of glacial and postglacial history. Species associated with these habitats may exhibit population patterns that mirror the postglacial history of the habitat. The Yukon grasslands have been hypothesized to be primarily composed of species that persisted in the Pleistocene Beringian steppe; or species that dispersed northward postglacially due to expanding prairie grasslands which later became disjunct because of mid-Holocene climate change.

Diptera populations found in all three grassland regions may have (1) survived glaciation as disjunct populations in Beringia and in the southern refugium; (2) dispersed northward postglacially from the southern refugium; or (3) arisen by a combination of the two. In terms of genetic expectations at the population level, each alternative would have different consequences: (1) would show distinct Yukon haplotypes in a network clearly separated from southern haplotypes; (2) would show haplotypes in the Yukon that are also found in the south, or are linked by short branches to clusters of southern haplotypes; (3) would show a combination of distinct clusters of Yukon haplotypes (reflecting survival and diversification in Beringia), as well as other Yukon haplotypes that are linked to clusters of southern haplotypes (reflecting additional dispersal of the populations northward postglacially).

Phylogeographic patterns for single species can be difficult to decipher due to factors such as mutation rates, gene flow within populations or variable genetic diversity through time [22]. Therefore, we selected three species of Diptera to examine congruence in patterns [23]. A major lineage within the order Diptera are brachyceran flies, or higher Diptera, which are stout flies with shortened antennae. This lineage includes acalyptrate flies, a polyphyletic grouping [24]. The study species were small (2–4 mm) acalyptrate flies which likely have limited dispersal abilities, a trait that could enhance genetic signals of past separation [25]. One of the species (*Incertella incerta* (Becker) (Chloropidae)) has a continuous distribution in multiple habitats, and two (*Trixoscelis fumipennis* (Melander) (Heleomyzidae) and *Meromyza columbi* Fedoseeva (Chloropidae)) are disjunct between the southern grasslands and the Yukon. By using species with different distribution patterns, we hoped to characterize the isolation which may have been due to range disjunction to ensure patterns were due to historical rather than landscape factors [22].

## Results

For each species, we obtained 17–21 sequences per gene (cytochrome *c* oxidase subunit I (COI) and cytochrome *b* (Cyt *b*)) per region, except *T. fumipennis* in the Prairie region (13 COI, 15 Cyt *b* sequences) (Additional file 1). Because no major differences were found in separate analyses, all results presented are for the concatenated dataset.

### Haplotype and nucleotide diversity

Haplotype and nucleotide diversity were calculated for each species. Both *I. incerta* and *T. fumipennis* had the same overall number of haplotypes (35), and similar values of haplotype and nucleotide diversity (Table 1). Although they also had the same number of haplotypes in the Yukon, *I. incerta* had higher nucleotide diversity in the Yukon, and while *I. incerta* had the same number of haplotypes (14) in both the Prairies and Peace River, there were more *T. fumipennis* haplotypes in the Peace River region (18 versus 11).

**Table 1:** Haplotype (*h*) and nucleotide (π) diversity for each species, per region

Species	*I. incerta*	*M. columbi*	*T. fumipennis*
For all regions			
Overall π	0.00501	0.00206	0.00489
Overall *h*	0.943	0.908	0.954
No. of haplotypes	35	21	35
Prairies			
π	0.00256	0.00172	0.00376
*h*	0.936	0.867	0.962
No. of haplotypes	14	9	11
Peace River			
﻿π	0.00337	0.00131	0.0049
*h*	0.956	0.829	0.971
No. of haplotypes	14	7	18
Yukon			
π	0.00449	0.00183	0.00196
*h*	0.732	0.918	0.745
No. of haplotypes	8	10	8

Overall, *I. incerta* nucleotide diversity decreased from north to south, but haplotype diversity was similar in the two southern regions and decreased in the north. For *T. fumipennis*, nucleotide and haplotype diversity were highest in the Peace River region and lowest in the Yukon. The overall nucleotide diversity of *M. columbi* was much lower than that of the other species and nucleotide and haplotype diversity were highest in the north.

### Haplotype networks

**Fig. 1:**
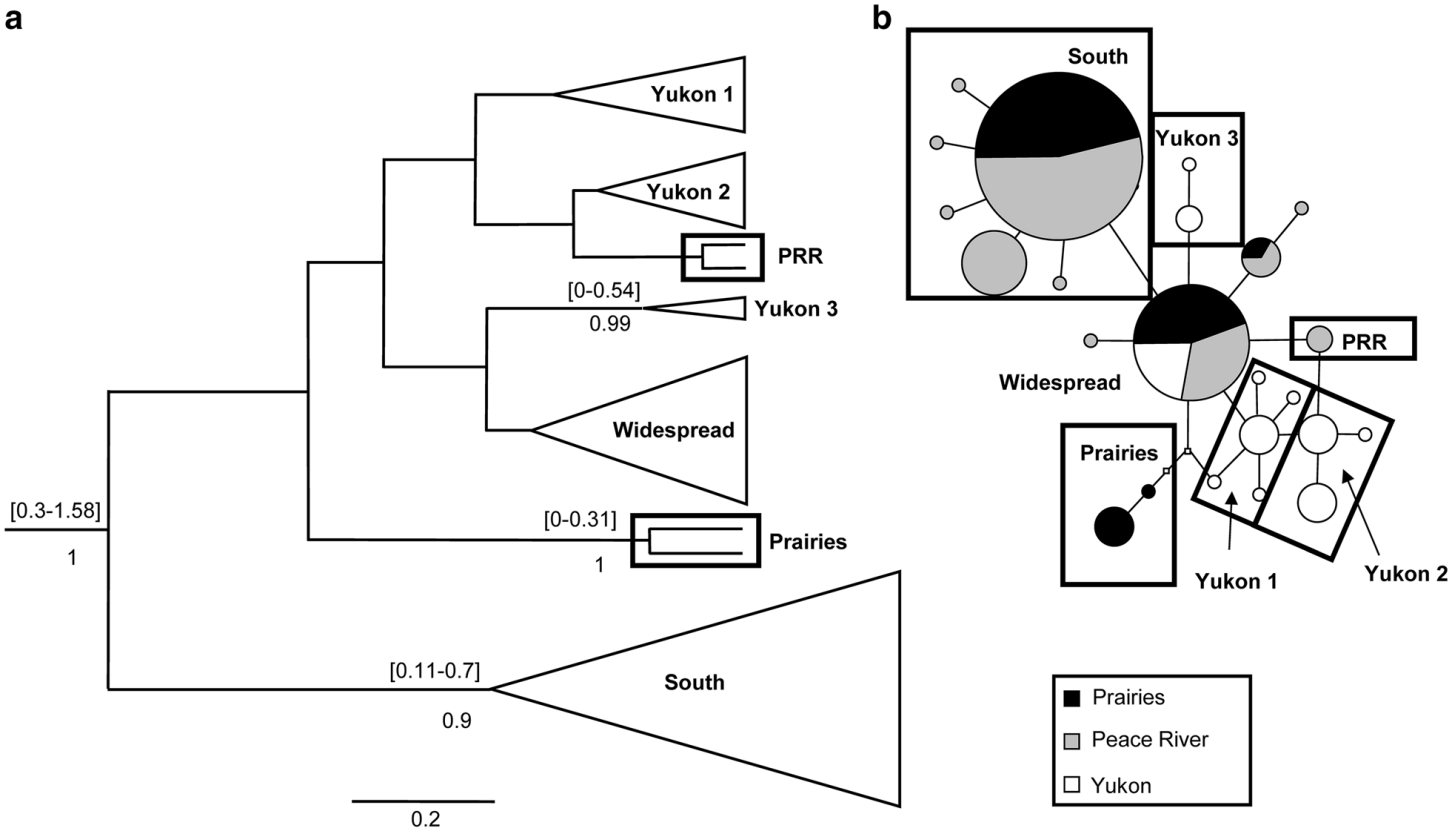
**a** Maximum clade credibility tree and **b** haplotype network of *Meromyza columbi* based on combined results from COI and Cyt *b*. **a** Posterior values of nodes below branch; 95% highest posterior density (HPD) interval of age (in Myr) of node above; branch lengths are scaled to time in Myr. **b** Each circle represents a single haplotype; small squares represent theoretical intermediates. Line lengths are arbitrary. Partitioned haplotypes represent haplotypes that are shared between regions. For the smallest circles, n = 1, otherwise size of circle is proportional to the abundance of the haplotype

Haplotype networks were built using statistical parsimony to characterize population patterns and examine them visually. Only *M. columbi* had a haplotype shared among all three regions (Fig. 1b). No haplotypes of *I. incerta* or *T. fumipennis* were shared between the two southern regions (Prairies + Peace River) and the Yukon.

**Fig. 2:**
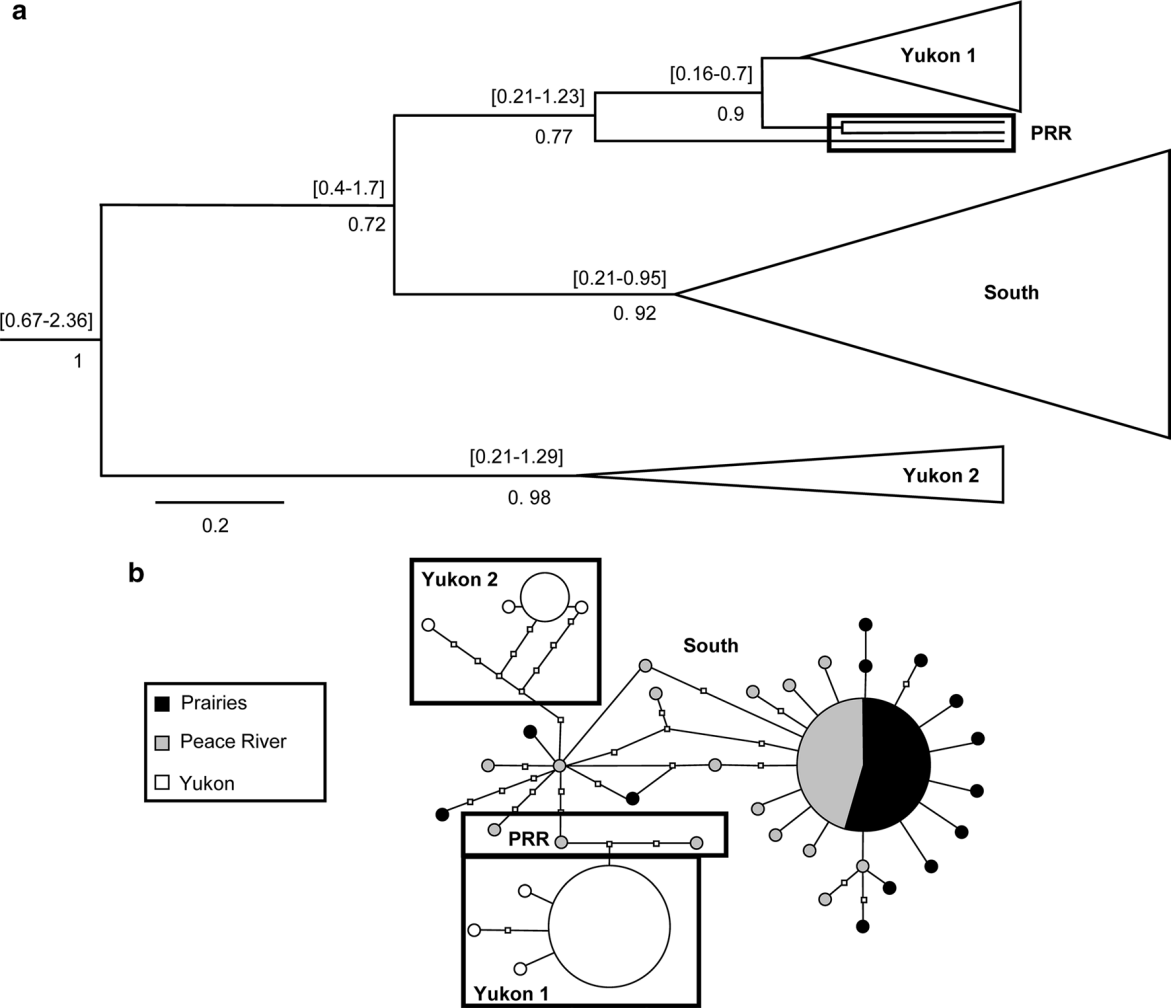
**a** Maximum clade credibility tree and **b** haplotype network of *Incertella incerta* based on combined results from COI and Cyt *b*. Symbols as in Fig. 1

**Fig. 3:**
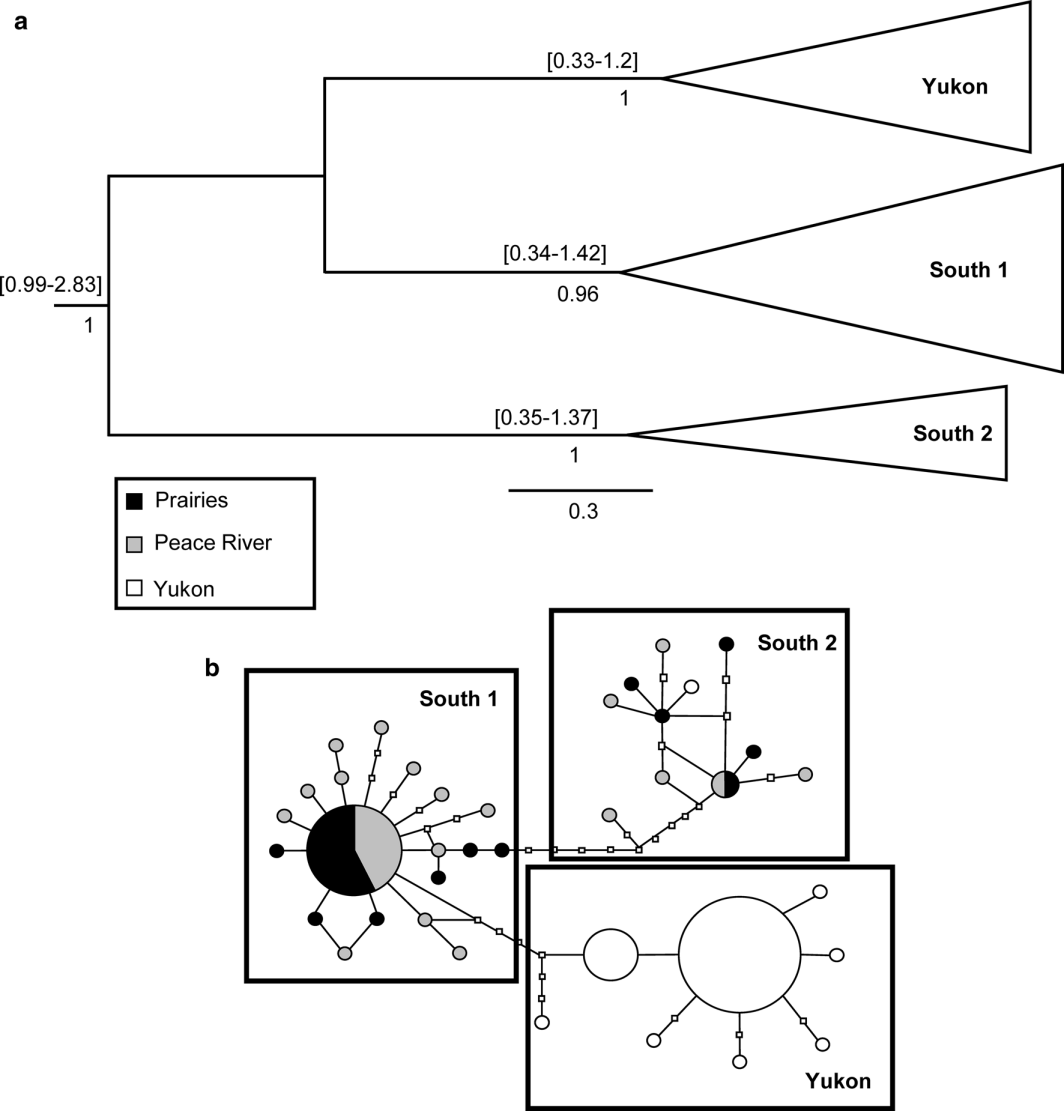
**a** Maximum clade credibility tree and **b** haplotype network of *Trixoscelis fumipennis* based on combined results from COI and Cyt *b*. Symbols as in Fig. 1

There were, however, shared haplotypes for each species between the two southern regions: *I. incerta* had one shared haplotype between the Prairies and Peace River region (Fig. 2b), *M. columbi* shared three (Fig. 1b) and *T. fumipennis*, two (Fig. 3b).

The two species with widespread southern geographic components, *I. incerta* and *T. fumipennis*, showed similarities in their networks not exhibited by *M. columbi*. Both had fewer, more common clustered Yukon haplotypes: *I. incerta* had two clusters, one of which was separated from other haplotypes by at least six base pair differences; and *T. fumipennis* had one, separated from other haplotypes by at least five base pair differences. A unique feature of the *T. fumipennis* network was the lone Yukon haplotype in a cluster of southern haplotypes (South 2, Fig. 3b).

In the *M. columbi* network, all haplotypes were separated by 1–2 base pair differences and there were fewer more common haplotypes. Despite links to haplotypes of other origins and one shared haplotype, the *M. columbi* Yukon haplotypes formed two clusters.

### Population structure and migration

We estimated the number of populations in our dataset without a priori assumptions using a Bayesian approach in the program Geneland [26]. Multiple runs in Geneland were consistent for each species. For *M. columbi* and *T. fumipennis*, two geographically structured populations were found: one Peace River + Prairie, and one Yukon. However, although *I. incerta* individuals grouped into the same geographically structured populations, Geneland recognized three populations. The third “ghost population”, which contained no individuals, was likely an artefact of the Bayesian analysis overestimating the genetic structure due to isolation by distance or data that did not adhere to modelling assumptions [27]. Guillot [27] recommended ignoring these ghost populations and Frantz et al. [28] suggested testing for isolation by distance. As expected, the partial Mantel test showed that *I. incerta* did exhibit isolation by distance, but population structure was also correlated with genetic distance when removing the effect of geographic distance (p = 0.06, R = 0.77), implying that population disjunction also plays a role in forming this pattern. The two other species overall did not show isolation by distance (results not shown).

We used analysis of molecular variance (AMOVA) to test for differentiation among the populations defined by Geneland. The AMOVA showed no significant differentiation between the southern population grouping of the Peace River and Prairie regions, and a Yukon population, for any species (p ~ 0.3, for all Φ_CT_) (Table 2).

**Table 2:** Results of AMOVA testing the structure outlined by Geneland

Species	Variance components			Percentage variation			Φ_CT_	p
	AP	AR/WP	WR	AP	AR/WP	WR		
*I. incerta*	1.707	0.051	2.00	45.32	1.37	53.32	0.453	0.3
*M. columbi*	0.475	0.028	0.919	33.40	1.97	64.63	0.334	0.3
*T. fumipennis*	2.012	− 0.027	1.950	51.14	− 0.70	49.56	0.511	0.3

One population contained all Prairie and Peace River individuals, the other all the Yukon individuals. *AP* among populations, *AR/WP* among region within populations, *WR* within region, *Φ*_*CT*_ fixation index of covariance among defined populations

An exact test of population differentiation (ETPD) was performed on this same population structure. The ETPD results were not concordant with AMOVA; there was a significant difference between the southern population assemblage (Peace River + Prairies) and the Yukon population (Table 3). In addition, comparisons among the two southern assemblages were not significant, supporting the population delimitations suggested by Geneland.

**Table 3:** Results from exact test of population differentiation and M values for each species

Species	R–PR		R–YK		PR–YK		South–YK
	*p* value	M	p-value	M	p-value	M	p-value
*I. incerta*	1.000	12.46	*> 0.0001*	0.52	*> 0.0001*	0.85	> 0.0001
*M. columbi*	0.112	13.53	*> 0.0001*	1.10	*0.00103*	0.85	*> 0.0001*
*T. fumipennis*	1.000	(infinite)	0.00571	0.39	*0.00323*	0.49	*0.00151*

Differentiation was examined for each pair of regions, as well as for the structure established by Geneland. Values in italic indicate significant p-values (p > 0.05). *R* Prairies, *PR* Peace River region, *YK* Yukon, *South* includes the Prairies and Peace River. M values (number of absolute migrants between regions per generation) are also included for each pair of regions

Migration was estimated using M values (absolute value of migrants between populations per generation). The M values showed that migration between the Peace River region and Prairies was high for all species, especially *T. fumipennis* whose M value was estimated to be infinity (Table 3). Although unrealistic, this value stems from an F_st_ value approaching 0, meaning that there is no differentiation between the two regions. In contrast, migration between those two regions and the Yukon was low for all species.

### Estimates of divergence time between populations

Dated Bayesian phylogenies were produced for each species to establish divergence times. Trees recovered groupings found in haplotype networks but did not resolve uncertain relationships. Clusters, particularly those from a single region of origin, tended to be supported in both networks and trees (e.g., Yukon 2 in the *I. incerta* tree) (Fig. 2). Unresolved regions in the haplotype networks were reflected in the Bayesian analyses through low posterior values (e.g., node of group Yukon 1, Yukon 2 and PRR in *M. columbi* tree) (Fig. 1).

The method used to find divergence times yielded conservative time intervals for nodes spanning hundreds of thousands and sometimes more than a million years. Furthermore, it is difficult to provide estimates for the divergence of Yukon populations from southern ones. Yukon individuals were not always assigned to the same monophyletic grouping (e.g., Yukon 1, 2 and 3 in *M. columbi* tree, Fig. 1a). In addition, branches at divergence nodes often had low posterior values (e.g., node of the group containing Yukon 1, Yukon 2, PRR in *M. columbi* tree, Fig. 1a). Posterior values below 0.7 are not reported. Nevertheless, nodes at the base of groupings (which represent the group’s time to most recent common ancestor (Tmrca, in million years (Myr)) with high posteriors allows placement of the origin of the group within a time frame. *Incertalla incerta* (Fig. 2a) and *T. fumipennis* (Fig. 3a) have nodes at the base of Yukon groups with high posteriors which range from well before the beginning of the Wisconsinan glacial, which began approximately 120 kya, and from before or during the Pleistocene glaciations (which began approximately 3 Mya [29]). The highest posterior density (HPD) interval of expansion for the Yukon population of *T. fumipennis* was 0.33–1.2 Mya (mean = 0.83) (Fig. 3a). For *I. incerta,* one of the Yukon groupings (Yukon 1) had an HPD interval between 0.16 and 0.7 Mya (mean = 0.67), and the Yukon 2 grouping 0.21–1.29 Mya (mean = 0.98) (Fig. 2a).

For *M. columbi*, only one of the nodes of a Yukon grouping had a high posterior (Yukon 3), with an HPD interval between 0 and 0.54 Mya (mean = 0.38) (Fig. 1a).

## Discussion

### Survival in Beringia, northward dispersal, or both?

Phylogeographic studies have shown that Beringia has been a refugium for species in multiple taxa, including plants, mammals and fish [5]. Our results mirror, at the population level, the patterns we found at the species level [21], in that widespread Diptera species in xeric Yukon grasslands are likely a mix of populations that apparently persisted in Beringian steppe communities through the Pleistocene, and those that dispersed northward during the Holocene. There was evidence for population differentiation between the Yukon and southern regions in some analyses (haplotype networks, ETPD, Geneland), although AMOVA results differed.

*Trixoscelis fumipennis* showed the most distinct division between Yukon and southern populations, with all Yukon haplotypes, except one, in a single group (Fig. 3b) whose divergence dates to the Pleistocene, based on Tmrca (Fig. 3a). This is suggestive of persistence of populations in Beringia during Pleistocene glaciation. The single Yukon haplotype in the South 2 group, otherwise containing only prairie and Peace region haplotypes, suggests a separate dispersal northward postglacially.

Haplotype networks and Bayesian analyses for *I. incerta* and *M. columbi* do not show such a clear pattern of population divergence.

In the two Yukon clusters of *Incertella incerta* (Fig. 2), the time intervals at the base overlap (0.16–0.7 vs. 0.21–1.29). It is possible that they originated at the same time and that additional sampling would recover missing intermediate haplotypes. However, the low haplotype diversity in the Yukon suggests that this is unlikely (Table 1). The number of base pairs separating the Yukon 2 cluster from the others accounts for high nucleotide diversity in this region (Fig. 2b), and may reflect a longer history of divergence, supported by the branch length of the Yukon 2 group (Fig. 2a).

The branches preceding the Yukon 1 group in *I. incerta* are short and nodes have low posterior values. Although this could be due to conflicting phylogenetic signal, this pattern is seen during rapid diversification events in species trees [30]. A rapid expansion event in an intraspecific tree would likely show the same signal. It is thus likely that this group has a more recent history.

This overall pattern is consistent with survival of populations of *I. incerta* in Beringia during the Pleistocene. While the region remained unglaciated, multiple glacials and interglacials affected species ranges, creating bottlenecks during glacials and allowing expansion during interglacials. The genetic patterns of *I. incerta* suggest that it could have been affected by at least two such events. The few frequent haplotypes in each group suggests that both were subjected to bottlenecks, and the branch lengths connected to each group suggest that they were affected by bottleneck events at different times.

Some phylogeographic studies have found population substructure within Beringia [5], as we did with *I. incerta*. In the ground squirrel, *Spermophilus parryii* Richardson (Rodentia: Sciuridae) at least four clades whose divergences can be dated back to glaciation events have persisted through several glacials [31]. The spruce beetle, *Dendroctonus rufipennis* Kirby (Coleoptera: Curculionidae), also exhibits two distinct clades in Beringia separated by a more southern clade which suggests secondary contact between both northern clades following glacial cycles [32].

In the case of our study, the precise reason for a population substructure in the *I. incerta* Yukon groups is unclear. The geographic extent of our sampling was limited and does not allow us to determine whether this substructure could be due to populations residing in different refugia, for instance. Many patterns of Beringian population substructure have been detected in different taxa [5] and our results warrant future investigation.

The haplotype network and dated tree of *M. columbi* are also difficult to interpret (Fig. 1). Few nodes have high posteriors and the HPD interval of 0–0.54 at the base of the Yukon 3 group encompasses the Holocene and the late Pleistocene. The low posterior values of the nodes and short branches preceding groups Yukon 1 and Yukon 2 suggest a period of rapid change, but it is difficult to speculate beyond this. Nonetheless, the high haplotype diversity in the Yukon, coupled with few shared haplotypes with the South, suggests survival of populations in Beringia [8]. Haplotype diversity of *M. columbi* was higher in the Yukon than in the other regions (Table 1), a pattern often associated with survival in a refugium [8], although this was not the case with the other species. The single widespread haplotype of *M. columbi* found in all three regions (Fig. 1b) suggests recent dispersal, consistent with a pattern seen in *I. incerta*.

Although results suggested that populations of all three species survived in Beringia during the Pleistocene, patterns differed considerably between species. This is not surprising given the differences in geographic distributions of each species, and their trophic roles. The lower haplotype and nucleotide diversity for the phytophagous species *M. columbi*, which has more disjunct populations compared to the other species, is consistent with patterns in butterflies with disjunct versus widespread distributions in the prairies and Peace River regions [22]. In such species, the distribution of suitable host plants is a factor in determining distribution. This may not be the case for generalist saprophagous species such as *I. incerta* or *T. fumipennis*. The more obvious genetic split between Yukon and southern populations of *T. fumipennis* mirrors the apparent disjunction in its overall distribution: it is widespread in southern Canada and the western United States but Foster and Mathis [33] recorded no specimens between prairie grassland sites in western Canada (British Columbia, Alberta, Saskatchewan) and the southern Yukon.

There are a few possible explanations for the disagreement between AMOVA (no significant population structure) and ETPD (significant difference in population structure). When examining disjunct and continuous butterfly species, Bromilow and Sperling [22] found that species with continuous distributions tended to lack significant population structure. This would correspond well with the distribution of *I. incerta* and it could be that the distributions of the other two species have not been recorded adequately. However, this would contradict ETPD results. The non-significant AMOVA could also be an artefact of small sample size. While this may be true particularly for *T. fumipennis*, where there was lower sampling in the Prairies, the sampling for *M. columbi* seems to have been sufficient given the higher frequency of many haplotypes. Another possibility is that the non-significance is due to the high variance both among population and within region, particularly for *I. incerta* and *T. fumipennis*. Certain haplotypes, even within populations or regions, are highly differentiated with many base pair differences in both the south and the Yukon. The presence of two Yukon groups of *I. incerta* haplotypes indicates population substructure which, among other explanations, could be due two different glaciation events.

Bromilow and Sperling [22] evaluated the population structure of continuously distributed and disjunct butterfly species in the Peace River and southern grasslands. Unlike their study, we did not find any significant population structure in the two southern regions. We also found more gene flow between both regions than Bromilow and Sperling [22] found in butterflies. The M values between the Peace River and Prairies were considerably higher (> 12.5) for all of our species than those found for any of the continuous species in their study (highest value: 10.24; average: 4.71). This was unexpected, because acalyptrate Diptera are thought to be poor fliers. One possible explanation for the gene flow between individuals from the Peace River and Prairies is that because of their small size acalyptrate flies can be passively dispersed over long distances by wind (e.g., [34, 35]). It may also be that populations of the Diptera species remained larger through time, and/or experienced fewer bottlenecks than Lepidoptera, and thus have retained higher genetic polymorphism.

Divergence time estimates are critical in testing phylogeographic hypotheses [36], but this is not a simple matter in Diptera, particularly Schizophora. Although strict-clock models tend to be appropriate for intraspecific datasets such as ours, the rate of evolution is a required parameter [37, 38]. Rates are known for mitochondrial genes of some Diptera, but individual Schizophora families exhibit substantially different diversification rates [24]. In addition, mutation rates may be time-scale dependent, where recently derived rates (through pedigree and laboratory studies) do not necessarily reflect rates on relevant time scales [39].

Because of the Pleistocene-Holocene time frame of our study, recent calibration points would have provided more accurate rate estimates for divergence time [40]. However, the massive Tertiary diversification of Schizophora has masked more recent phylogenetic patterns, and existing deep fossil calibration points for flies are not specific to our study taxa [24]. In addition, deep calibration times are problematic beyond time-dependency biases. Divergence in genes often predates population divergence and this can also lead to an overestimate of divergence times.

To mitigate issues regarding time-dependency due to calibration constraints, we used multiple demographic models depending on whether the data was interspecific or intraspecific [41]. Other methods have been suggested to deal with these issues, such as expansion dating where a well-documented population expansion was used to calibrate rates [42]. However, the lack of reliable data on phylogeny, diversity and population patterns in many Diptera limits the possible approaches to obtaining divergence time estimates. Although it is possible that our method has inflated divergence times, our estimates do fit the time frame of the Pleistocene glaciations [29].

While our analyses are concordant with one another, this study was based on two mitochondrial genes, which represent only the matrilineal side and may not fully reflect population history [43]. Additional mitochondrial or nuclear genes could provide more insight into these patterns.

## Conclusions

Our analyses support the conclusion that populations of Diptera in Yukon grasslands could have persisted in steppe habitats in Beringia through Pleistocene glaciations. Current populations in the region appear to be a mix of Beringian relict populations and, to a lesser extent, postglacial dispersal northward from southern prairie grasslands.

Given the limited current extant and potential glacial history of xeric Yukon grasslands, they have been surprisingly understudied. Most research to date has been focused on paleoecological data and current species assemblages, and not on phylogeography or genetic patterns (e.g., [21, 44–46]). In a comparison of plant species present in Alaska, in the Boreal forest on the northern Great Plains, and the southwest Yukon, Vetter [45] found that 25% of plant species in each region were restricted to that region. These grasslands are not uniform in their composition and could potentially have different origins or at least be good systems in which to study modern landscape genetics if these differences are recent. These grasslands offer a unique opportunity to study ice age dynamics through extant systems.

Most phylogeographic work with a Beringian scope has been on arctic and alpine organisms (e.g., most examples in Shafer et al. [5]). However, xeric Beringian grasslands may be as vulnerable to climate change as other Arctic ecosystems. Conway and Danby [47] found a reduction in the extent of grassland due to forest encroachment, particularly on flat terrain and south-facing slopes near Kluane Lake, Yukon. Although more restricted than other arctic ecosystems, these grasslands host a unique species assemblage. Some insects, such as the weevil *Connatichela artemisiae* Anderson (Coleoptera: Curculionidae), are endemic to eastern Beringian grasslands [48], and some potentially endemic Diptera await formal description (A.M. Solecki and J.J. Mlynarek, unpublished data). Our study has shown that flies present on these south-facing slopes represent unique genetic lineages. The insect fauna of these grasslands may be as distinct and unique as the grasslands themselves.

## Methods

### Sampling sites

**Fig. 4:**
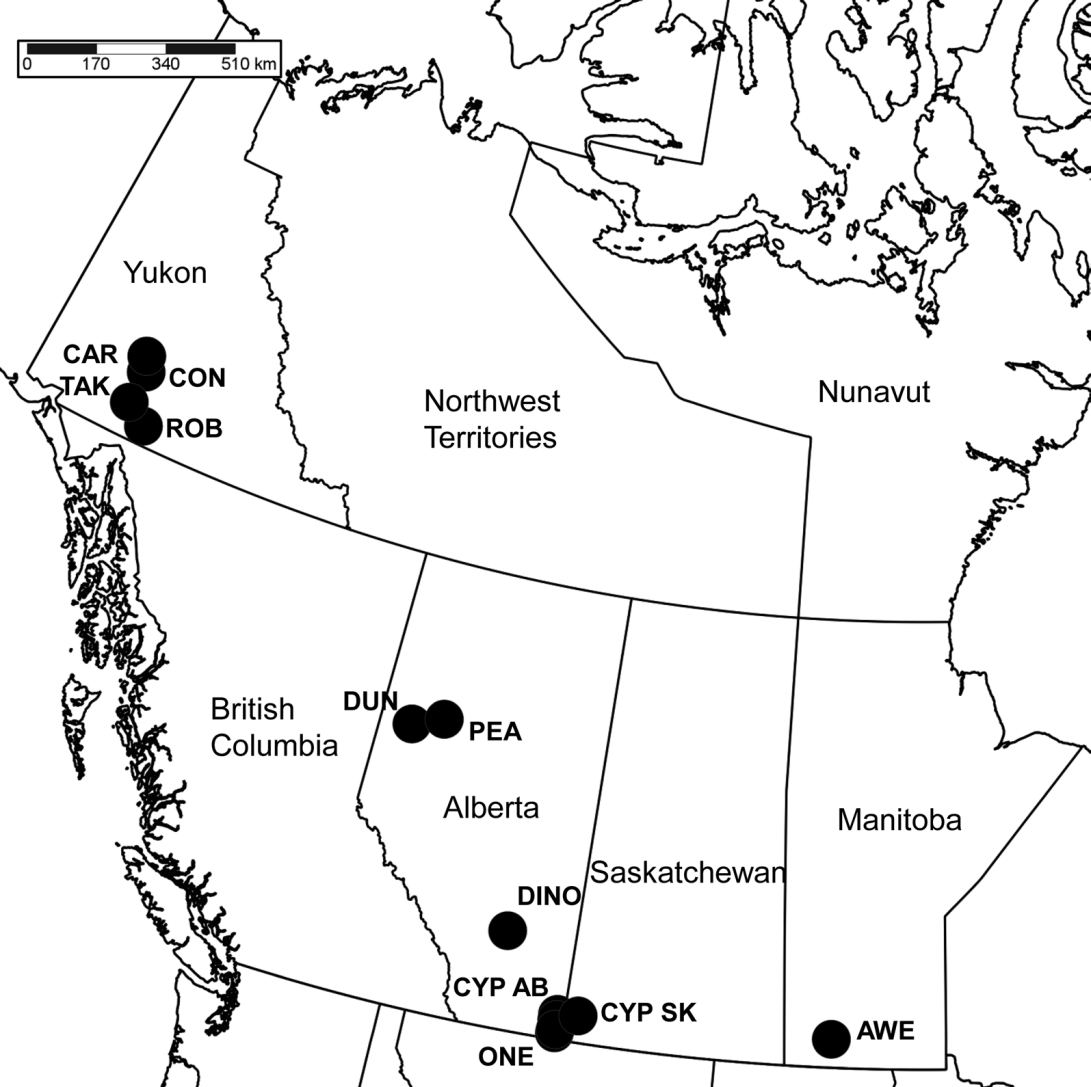
Map (Lambert projection) of sampling localities. Site codes: *AWE* Aweme, *CAR* Carmacks, *CON* Conglomerate, *CYP AB* Cypress Hills AB, *CYP SK* Cypress Hills SK, *DINO* Dinosaur, *DUN* Dunvegan, *ONE* Onefour, *PEA* Peace, *ROB* Robinson, *TAK* Takhini. Map created with SimpleMappr [51]

Diptera were collected from grasslands in three regions in Canada: Prairies (Alberta, 5 sites; Saskatchewan, 1 site; Manitoba, 2 sites), the Peace River region (Alberta, 2 sites), and southern Yukon Territory (4 sites) (Table 4, Fig. 4).

**Table 4:** Sampling locations and their coordinates divided by designated region

Location	Abbreviation	N	W
Prairies region			
Onefour Heritage Land, AB	Onefour	49.15666	− 110.2635
Cypress Hills Interprov. Park, AB	Cypress Hills AB	49.6291	− 110.2623
Rte 41, South of Cypress Hills Interprov. Park, AB	Cypress Hills AB	49.49087	− 110.2547
Rte 41, near Cressday, AB	Onefour	49.2444	− 110.2525
Midland Provincial Park, McMullen Island, near Dinosaur Trail, AB	Dinosaur	51.46943	− 112.771
Cypress Hills Interprov. Park, SK	Cypress Hills SK	49.6718	− 109.4615
Aweme/Criddle Vane Homestead Prov. Park, MB	Aweme	49.70853	− 99.60275
Peace River region			
Dunvegan, AB	Dunvegan	55.92543	− 118.59856
Peace River, Grouard Hill, AB	Peace	56.2325	− 117.2770
Southern Yukon			
Robinson Road House, YT	Robinson	60.44839	− 134.84961
Conglomerate Mtn, 8.8 km South of Twin Lakes, YT	Conglomerate	61.6273	− 135.8802
Bushy Mountain, 15 km South of Carmacks, YT	Carmacks	61.9700	− 136.2033
13.1 km west of Takhini River, YT	Takhini	60.81407	− 135.9706

Vegetation in the Prairies is broadly characterized by grasses (Poaceae), sedges (Cyperaceae), Asteraceae, especially sages (*Artemisia*), and other forbs [20]. Sampling focused on dry sites in the mixed grassland ecoregion, dominated by blue grama, speargrass, low sedge and *Artemisia frigida* [49]. Sites characterized by different vegetation (e.g., tall-grass prairie, Cypress Uplands) were also sampled for widespread Diptera species.

The Peace River grasslands are isolated from the southern Prairies by 300–400 km and are restricted to the Peace River valley and its tributaries [19]. Sampling in the Peace River was restricted to xeric, steep slopes which tend to have *Hesperostipa spartea*-*Carex*-*A. frigida* associations [50].

Yukon grasslands are characterized by *A. frigida*, bunch grasses and forbs and are generally associated with arid, exposed south-facing slopes [16].

### Taxonomic sampling

**Fig. 5:**
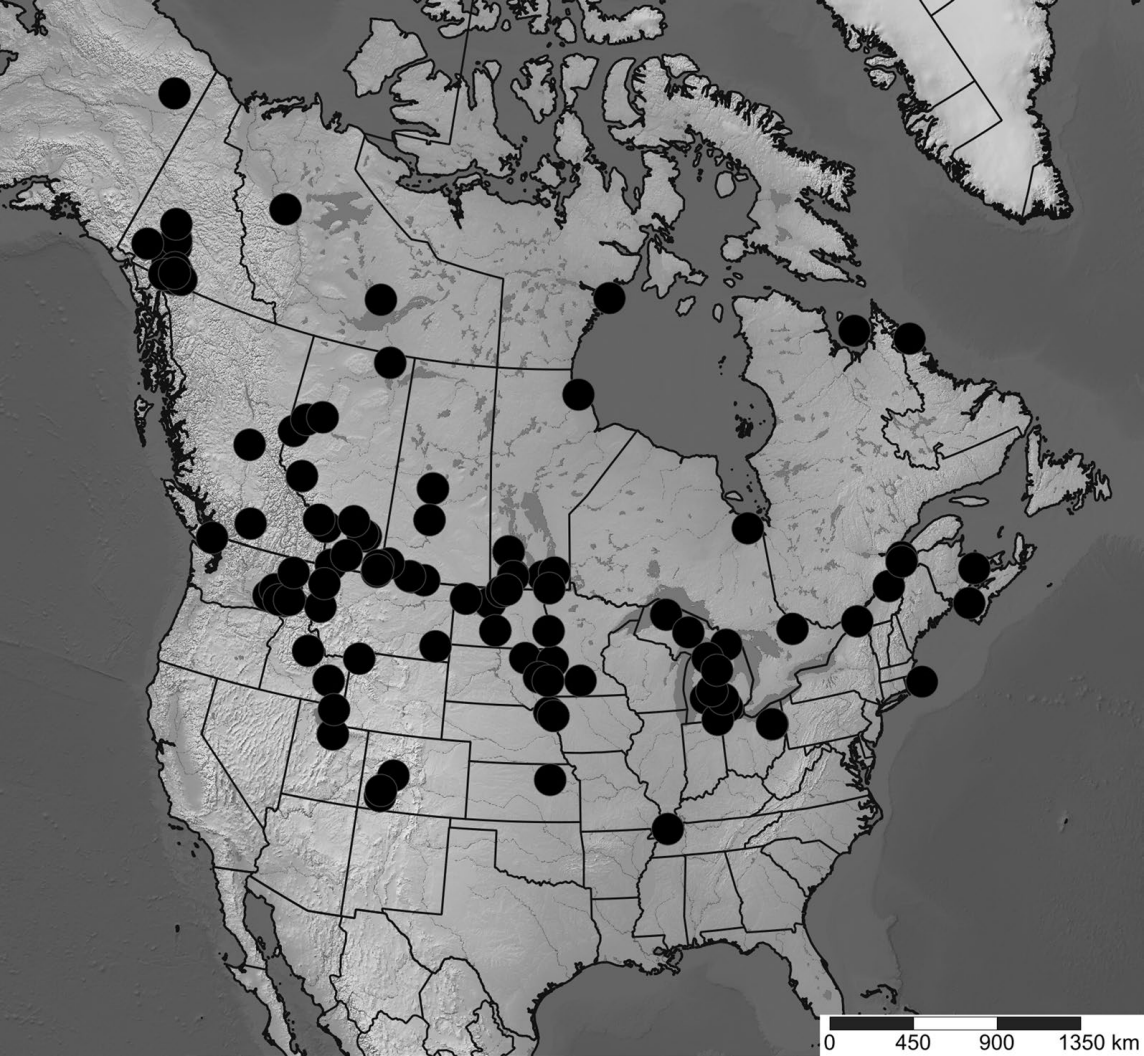
Distribution map (Lambert projection) of *Incertella incerta*. Map based on records from databases (Canadensys [53], BOLD [54]), Lyman Entomological Museum (not yet databased in Canadensys) or other literature. Coordinates in Additional file 2. Map created with SimpleMappr [51]

**Fig. 6:**
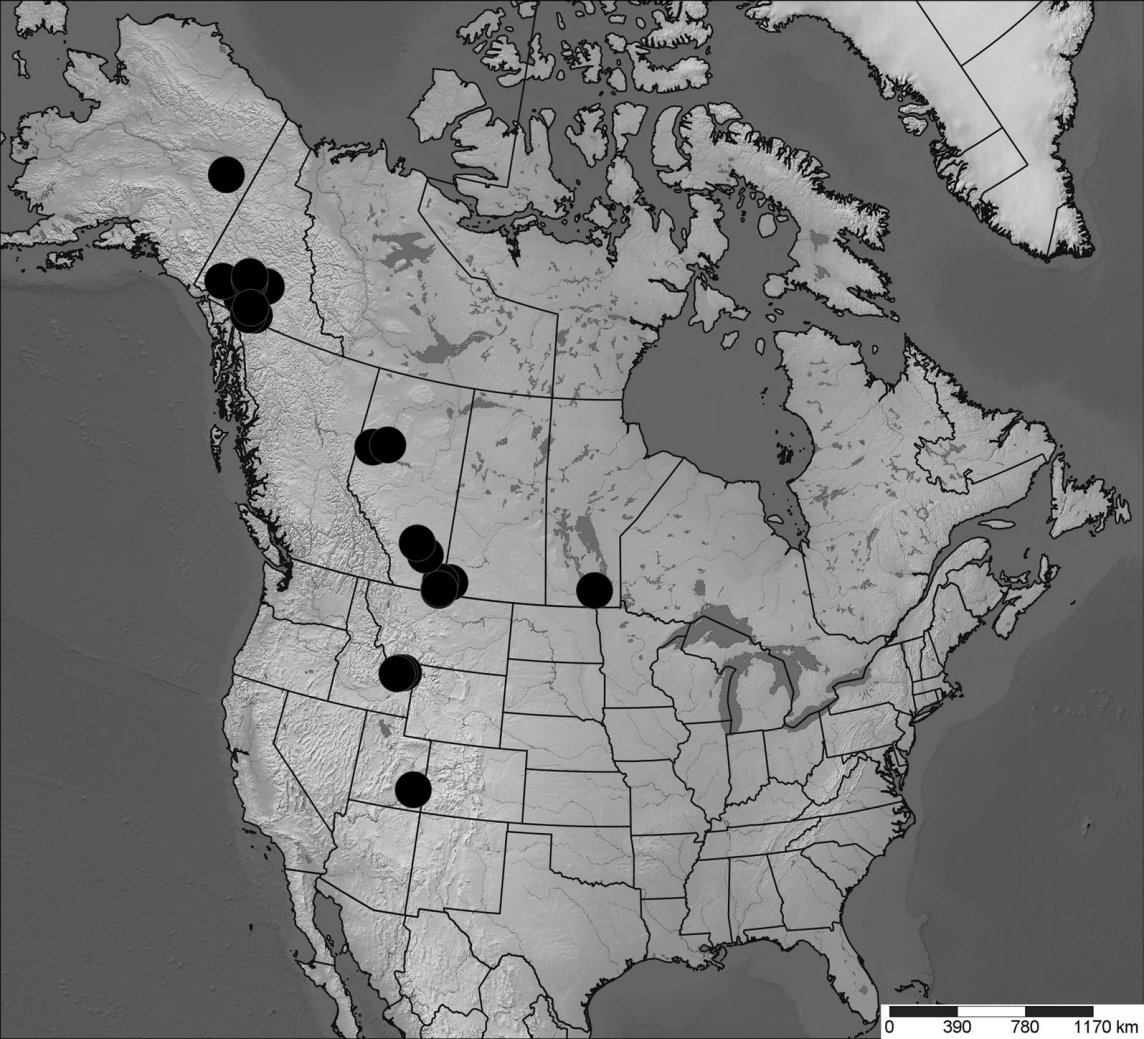
Distribution map (Lambert projection) of *Meromyza columbi*. Map based on records from Canadensys [53], BOLD [54], Lyman Entomological Museum (not yet databased in Canadensys) or Fedoseeva [52]. Coordinates in Additional file 2. Map created with SimpleMappr [51]

**Fig. 7:**
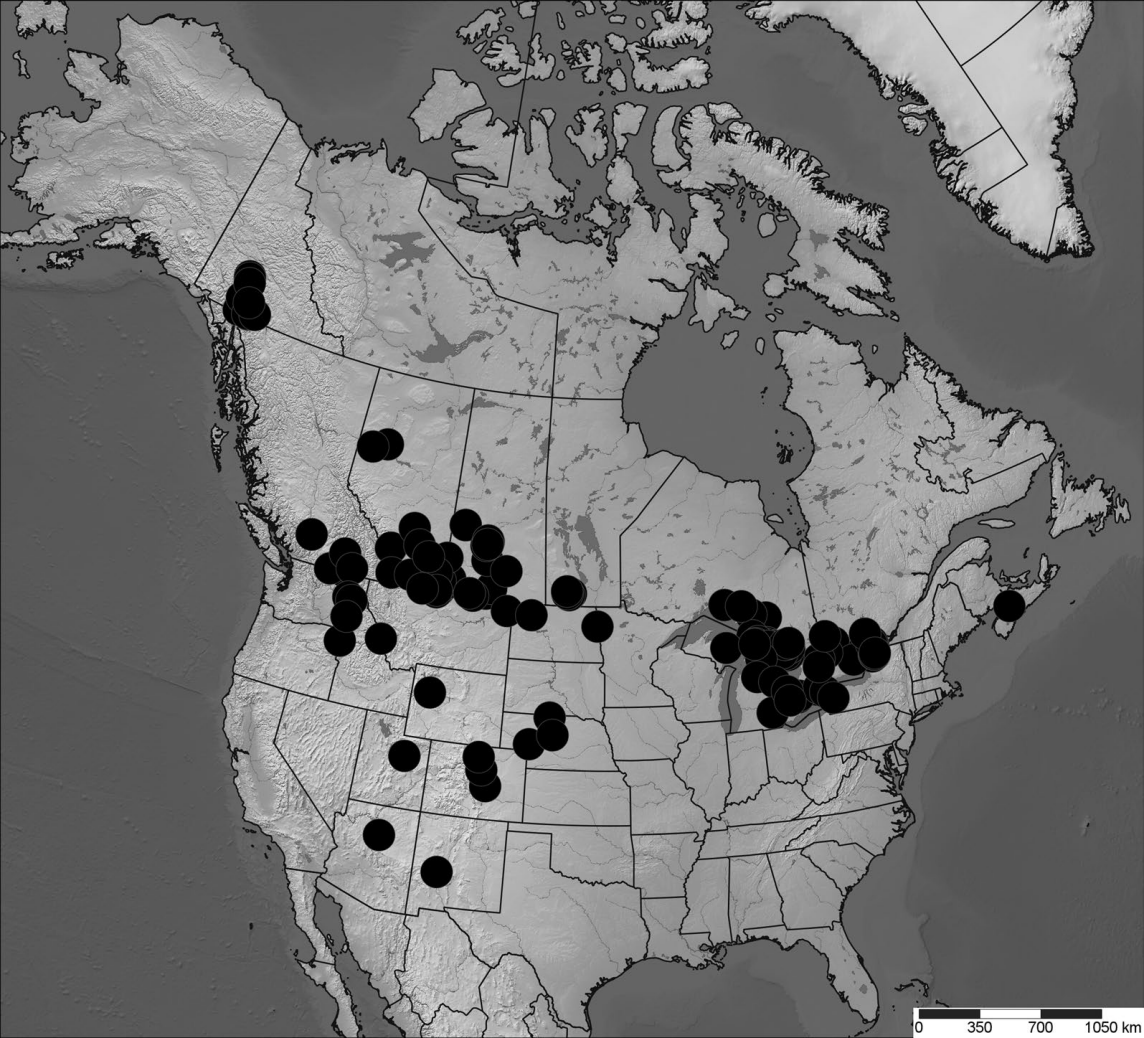
Distribution map (Lambert projection) of *Trixoscelis fumipennis.* Map based on records from Canadensys [53], BOLD [54], Lyman Entomological Museum (not yet databased in Canadensys) or Foster and Mathis [33]. Coordinates in Additional file 2. Map created with SimpleMappr [51]

Diptera were collected into 95% ethanol and dried with hexamethyldisilazane prior to mounting. Specimens were identified to species to determine those present in all three regions. Geographic distributions were determined by creating maps [51] using published literature [33, 52] and museum records [53, 54] (Additional file 2). Three species were selected for analysis: *Incertella incerta* (Becker) (Chloropidae), a widespread Nearctic generalist saprophagous species, present in habitats between the study regions (Fig. 5); *Meromyza columbi* Fedoseeva (Chloropidae), a phytophagous western Nearctic grassland species disjunct between the three study regions [52] (Fig. 6); and *Trixoscelis fumipennis* (Melander) (Heleomyzidae), an apparently saprophagous species widespread in the southern Nearctic, south of the Peace River region, but disjunct between Peace River and the Yukon (Fig. 7). Although *T. fumipennis* is mostly present at disturbed sites in its southern range [33], in the Yukon it has been collected almost exclusively in xeric grassland sites.

### Molecular techniques

DNA extraction, amplification and sequencing protocols follow Gibson et al. [55]. Total genomic DNA was extracted using whole specimens. For each species, 20–21 specimens were extracted for each region. Since specimens were mounted on points, water was used to dissolve glue prior to extraction when necessary. The DNA was extracted using a DNeasy Tissue kit (Qiagen Inc., Santa Clara, CA, USA). After extraction, specimens were critical point dried. All specimens were assigned unique identifiers and vouchers are deposited at the Lyman Entomological Museum, McGill University (Table 5).

**Table 5:** Voucher identifiers and GenBank accession numbers for CO1 and Cyt *b*, by species and location

Location	Unique identifiers	Accession COI	Accession Cyt *b*
*Meromyza columbi*			
Onefour	LEM0276023	KP037115	KP037303
	LEM0276024–27	KP037169–172	KP037356–9
	LEM0276028	–	KP037355
	LEM0276029–30	KP037167–8	KP037353–4
	LEM0276136	KP037146	KP037332
	LEM0276261–2	KP037118–9	–
	LEM0276263–4	KP037116–7	KP037304–5
Cypress Hills AB	LEM0276047	KP037150	KP037336
	LEM0276255	KP037124	KP037310
Dinosaur	LEM0276257–60	KP037120–3	KP037306–9
Dunvegan	LEM0276031–8	KP037159–66	KP037345–52
	LEM0276204	KP037145	KP037331
	LEM0276245–54	KP037125–34	KP037311–20
Peace	LEM0276134–5	KP037147–8	KP037333–4
Takhini	LEM0276040	KP037157	KP037343
	LEM0276045–6	KP037151–2	KP037337–8
	LEM0276133	KP037149	KP037335
	LEM0276235–44	KP037135–44	KP037321–30
Carmacks	LEM0276039	KP037158	KP037344
	LEM0276041–4	KP037153–6	KP037339–42
*Incertella incerta*			
Onefour	LEM0276048	KP037106	KP037293
	LEM0276059–60	KP037087–8	KP037273–4
	LEM0276061	–	KP037275
	LEM0276062	KP037089	KP037276
	LEM0276216	KP037070	KP037256
	LEM0276220–2	KP037064–6	KP037250–2
	LEM0276224	KP037062	KP037248
Cypress Hills AB	LEM0276049	KP037107	KP037294
	LEM0276215	KP037071	KP037257
	LEM0276217–9	KP037067–9	KP037253–5
	LEM0276223	KP037063	KP037249
Cypress Hills SK	LEM0276055–7	KP037108–10	KP037295–7
	LEM0276058	KP037082	KP037268
Dunvegan	LEM0276063–65	KP037092–94	KP037279–81
	LEM0276067–70	KP037096–99	KP037283–86
	LEM0276075	KP037104	KP037291
	LEM0276139–40	KP037083–4	KP037269–70
	LEM0276225	KP037061	KP037247
	LEM0276226	–	KP037246
	LEM0276227–28	KP037059–60	KP037244–5
	LEM0276229	–	KP037302
	LEM0276231–34	KP037111–4	KP037298–301
Carmacks	LEM0276208	KP037078	KP037264
Robinson	LEM0276066	KP037095	KP037282
	LEM0276071–4	KP037100–3	KP037287–90
	LEM0276076	KP037105	KP037292
	LEM0276077–8	KP037090–1	KP037277–8
	LEM0276137–8	KP037085–6	KP037271–2
	LEM0276205–7	KP037079–81	KP037265–67
	LEM0276209–14	KP037072–7	KP037258–63
*Trixoscelis fumipennis*			
Aweme	LEM0049920	KP037201	KP037388
	LEM0049922–3	–	KP037389–90
	LEM0276093	KP037215	KP037404
Cypress Hills AB	LEM0276094–6	KP037212–4	KP037401–3
	LEM0276099	KP037209	KP037398
	LEM0276132	KP037178	KP037365
Cypress Hills SK	LEM0276100	KP037208	KP037397
Onefour	LEM0276101–2	KP037206–7	–
	LEM0276131	KP037177	KP037364
	LEM0276285–6	KP037198–9	KP037385–6
Dunvegan	LEM0276054	KP037200	KP037387
	LEM0276087–92	KP037216–21	KP037405–10
	LEM0276097–8	KP037210–1	KP037399–400
	LEM0276127	KP037205	KP037394
	LEM0276273–4	KP037187–8	KP037374–5
Peace	LEM0276128	KP037204	KP037393
	LEM0276265–72	KP037179–86	KP037366–73
Robinson	LEM0276079–82	KP037173–6	KP037360–3
	LEM0276083–6	KP037222–5	KP037411–14
	LEM0276129–30	KP037202–3	KP037391–2
	LEM0276275–76	KP037189–90	KP037376–7
	LEM0276277	KP037191	–
	LEM0276278–83	KP037192–97	KP037378–83
	LEM0276284	–	KP037384

Vouchers are deposited at the Lyman Entomological Museum. Abbreviations as in Table 4

Two mitochondrial gene regions were targeted and amplified: (1) a 658 bp fragment of cytochrome *c* oxidase subunit I (COI) (the DNA barcode) using the forward primer LC01490 (5′-GGTCAACAAATCATAAAGATATTGG-3′) [56] and the reverse primer COI-Dipt-2183R (5′-CCAAAAAATCARAATARRTGYTG-3′) [57]; (2) a 510 bp fragment of cytochrome *b* using the forward primer CytB-Dipt-11035F (5′-GGNTTYKCNGTNGAYAAYGC-3′) [57] and the reverse primer CytB-Dipt-11545R (5′-ACDGGDCGDGCYCCRATTC-3′) [57]. Amplifications were carried out in 25 μL reactions: 16.75 μL ddH_2_O, 2.5 μL 10X Ex-Taq PCR buffer (containing 20 mM MgCl_2_), 0.625 μL 25 mM MgCl_2_, 1 μL of each 10 μM primer, 2 μL 10 μM dNTPs, 0.125 μL ExTaq HS DNA polymerase (Takara Bio USA, Madison, WI, USA), and 1 μL genomic DNA template. Amplification cycles were performed on an Eppendorf ep Gradient S Mastercycler (Eppendorf AG, Hamburg, Germany) as follows: 94 °C for 3 min; 30 amplification cycles of 94 °C for 45 s, 45 °C for 45 s, 72 °C for 1 min; and a final step for 5 min at 72 °C.

Amplification products were visualised on 1% agarose electrophoresis gels and target genes were isolated and purified using the E-Gel^®^ system (Invitrogen™, Carlsbad, CA, USA) as outlined in Gibson et al. [58]. Purified products were sequenced at the Agriculture & Agri-Food Canada, Eastern Cereal and Oilseed Research Centre Core Sequencing Facility (Ottawa, ON, Canada). The same primers used in PCR reactions were used to sequence forward and reverse strands. Sequencing reactions were carried out in a volume of 10 μL and used an ABI BigDye^®^ Terminator v3.1 Cycle Sequencing kit (PE Applied Biosystems, Foster City, CA, USA).

Chromatograms for sequences LEM0276023–0276140 were edited and visualised using Sequencher 4.7 (Gene Codes Corp., Ann Arbour, MI, USA). Other chromatograms (sequences LEM0049920, LEM0049922–0049923, LEM0276204–0276286) were edited and visualised using ChromasPro (Technelysium, South Brisbane, QLD, Australia).

Sequences were aligned using Clustal X v.2.0 with default parameters [59]. Overhangs were removed in BioEdit v.7.2.3 [60]. Nucleotide sequences were translated into amino acids using the invertebrate mitochondrial genetic code with ORF Finder [61] to place sequences in the appropriate reading frame. GenBank numbers for all sequences are in Table 5.

### Statistical analyses

All analyses were performed on the concatenated dataset. Mitochondrial haplotype diversity (*h*) and nucleotide diversity (π) were calculated for the entire dataset and for each region using the program DnaSP v.5 [62].

Haplotype networks were constructed using statistical parsimony with the program TCS v.1.2.1 [63] with a 95% cut-off value for parsimonious branch connections between haplotypes.

To avoid a priori assumptions about the data, the program Geneland v.4.0.3 [64] run in R [65] was used to estimate the number of populations in the total sample. It uses a Bayesian approach that can incorporate molecular and geographic data to estimate population clusters without prior population definitions [26].

The Geneland analysis was performed under the correlated allele frequencies model. Geographic coordinates (WGS84) were converted to UTM. Coordinate uncertainty did not affect results and thus, for the final runs, none was assigned. The analysis was executed for five runs, with 1,000,000 Markov chain Monte Carlo (MCMC) iterations each. Thinning was set at 400. The minimum and maximum value of K (number of populations) was set to 1 and 10, respectively. Burn-in was set to 2000.

Because Bayesian methods can overestimate population structure when there is isolation by distance [28], we examined correlation between geographic and genetic distance using partial Mantel tests executed in Isolation by distance web service v.3.23 [66]. The partial Mantel test compares genetic and geographic distances while allowing additional variables to be incorporated into the test and for their effects to be isolated [67]. We removed the effect of pre-existing population structure due to disjunction to verify that patterns were not just due to isolation by distance [22]. To build this indicator matrix, for each pairwise comparison, a value of 0 was given when both individual sequences came from the same population and 1 when they did not. For each species, 10,000 randomizations were performed. The genetic distances were calculated using Φ_ST_ and Kimura’s Two-Parameter Model which accounts for different rates of transition and transversion [68].

Population structures defined by Geneland were tested within an AMOVA framework and with ETPD. Both were implemented in Arlequin v.3.5 [69]. The AMOVA was examined at three hierarchical levels: Φ_ST_—within region (regions being defined as Prairies, Peace River region or Yukon), Φ_SC_—within regions among populations and Φ_CT_—among populations as defined by Geneland. Arlequin was also used to calculate M values using the formula M = (1 − F_ST_)/2F_ST_ [70].

### Divergence times

Divergence times were calculated with BEAST v.1.7.5 [71] and the output examined via Tracer v.1.6 [72]. Because there are no suitable recent calibration points or intraspecific mutation rates for the study taxa, we used fossil data to estimate mutation rates. A dated Diptera tree was calibrated using fossils for specific clades and dates obtained at the nodes of the relevant lineages were used for subsequent analyses. This method was adapted from Nardi et al. [73] and Marino et al. [41].

The Diptera tree was built using sequences of Cyclorrhapha, Schizophora and Acalyptratae obtained from GenBank (Additional file 3). The two fossil dating points used were: 70 Mya for Schizophora [24, 73] and 42 Mya for Chloropidae [24] (Table 6). The Hasegawa-Kishino-Yano (HKY) nucleotide substitution model was chosen for sequence evolution [74] with partition of nucleotides into their separate coding positions and rate variation described by a four category discrete distribution. Models that consider codons tend to outperform models that do not, even when fewer parameters are considered [75]. The two genes were unlinked to allow separate base frequencies to be estimated. A relaxed lognormal clock model was used to allow different rates of evolution for each branch [76]. The tree prior for this interspecific Diptera tree was set as the Yule process [77], a model appropriate for multiple species. MCMC chain length was set to 100 million, with a 10% burn-in. Convergence was confirmed by examining Effective Sample Size (ESS > 200). Other parameters were set to the default.

**Table 6:** Priors for calibrating phylogenies in BEAST

Analysis	Taxon	Distribution	Calibration type	Priors
Diptera phylogeny	Schizophora	Lognormal	Fossil [50, 52]	Mean = 3 St. dev. = 0.78 Offset = 70
	Chloropidae	Lognormal	Fossil [52]	Mean = 3 St. dev = 0.7 Offset = 42
Simplified Diptera Phylogeny	Chloropidae	Normal	Posterior distribution from Diptera phylogeny	Mean = 59.41 St.dev. = 8.9
	*Incertella incerta*	Normal	Posterior distribution from Diptera phylogeny	Mean = 31.93 St.dev = 11.54
	*Meromyza columbi*	Normal	Posterior distribution from Diptera phylogeny	Mean = 23.48 St.dev = 10.3
	*Trixoscelis fumipennis*	Normal	Posterior distribution from Diptera phylogeny	Mean = 29.3 St.dev = 13.14
Demography	*Incertella incerta*	Normal	Tmrca from simplified phylogeny	Mean = 1.788 St.dev = 0.63 μ = 0.0042661
	*Meromyza columbi*	Normal	Tmrca from simplified phylogeny	Mean = 1.32 St.dev = 0.47 μ = 0.0023813
	*Trixoscelis fumipennis*	Normal	Tmrca from simplified phylogeny	Mean = 2.23 St.dev = 0.64 μ = 0.0027401

Tmrca is the time to most recent common ancestor (in Myr) and μ is the mutation rate (in mutations per site per million year)

A simplified phylogeny of each study taxon was then generated, using one sequence per haplotype and the other Chloropidae species to root the tree. For each subset, the HKY model with invariant sites was selected based on results from jModelTest 2 (v.2.1.4) [78]. Nucleotides were partitioned into their separate coding positions. Posterior distributions from the Diptera tree were used as priors to calibrate the Chloropidae and the study taxa nodes (Table 6, Additional file 3). The intraspecific aspect of the data was ignored and a Birth–Death prior was used [41]. The analyses were performed under a strict clock for a MCMC chain length of 10 million (10% burn-in). A strict molecular clock was used for this and the following analyses due to the intraspecific nature of the data and the expected low rate of variation between branches [37]. Analyses were verified for convergence (ESS > 200).

Demographic analyses were run using BEAST, for each species separately. All sequences available for the study taxa were used. Tree shape was defined by a Bayesian skyline prior, a variable population size coalescent model [79]. The Tmrca, and mutation rate for each taxon from the simplified phylogenies were used as priors (Table 6). Analyses were run for a MCMC length of 30 million (ESS > 200) with a 10% burn-in. Maximum clade credibility trees were viewed in FigTree v.1.4.0 [80].

## Supplementary information

**Additional file 1.** Number of sequences per species per location. Abbreviations as in Table 4.

**Additional file 2.** Coordinates for distribution maps of all 3 species. Each excel sheet contains the coordinates used to create the distribution map of one of the species along with the locality information and source.

**Additional file 3.** GenBank accession numbers for Diptera phylogeny. Additional taxa (with GenBank accession numbers) used for calculating divergence times.

## Data Availability

The sequences used in this study have been deposited in GenBank under the accession numbers outlined in Table 5.

## Abbreviations

AMOVA: analysis of molecular variance
COI: cytochrome *c* oxidase subunit I
Cyt *b*: cytochrome *b*
ESS: effective sample size
ETPD: exact tests of population differentiation
*h*: haplotype diversity
HKY: Hasegawa-Kishino-Yano
HPD: highest posterior density
kya: thousand years ago
M value: absolute value of migrants between populations per generation
MCMC: Markov Chain Monte Carlo
Mya: million years ago
Myr: million years
Tmrca: time to most recent common ancestor (in millions of years)
π: nucleotide diversity
