# Correction to: Phylogeography of higher Diptera in glacial and postglacial grasslands in western North America

**DOI:** 10.1186/s12898-020-00302-w

**Published:** 2020-07-09

**Authors:** Anna M. Solecki, Jeffrey H. Skevington, Christopher M. Buddle, Terry A. Wheeler

**Affiliations:** 1 Department of Integrative Biology, University of Guelph, Guelph, ON N1G 2W1 Canada; 2 Natural Resource Sciences, McGill University, Macdonald Campus, Ste-Anne-De-Bellevue, QC H9X 3V9 Canada; 3 Agriculture and Agri-Food Canada, Canadian National Collection of Insects, Arachnids, and Nematodes, 960 Carling Ave, Ottawa, ON K1A 0C6 Canada

## Correction to: BMC Ecol (2019) 19:53 10.1186/s12898-019-0266-4

Following publication of the original article [1], the authors reported an error with the copyright holder. The incorrect copyright is: © The Author(s) 2019. The correct copyright holder is: © Crown 2019.

The original article [1] has been updated.
