# Supplementary material for: Phylogeography of higher Diptera in glacial and postglacial grasslands in western North America
Source: BMC Ecol. 2019 Dec 20;19:53. doi: 10.1186/s12898-019-0266-4 (PMC6923875; doi:10.1186/s12898-019-0266-4)
Supplement: Supplementary file 1 — Additional file 1. Number of sequences per species per location. Abbreviations as in Table 4. [file 12898_2019_266_MOESM1_ESM.docx]

**Additional file 1**: Number of sequences per species per location. Abbreviations as in Table 4.

| Species | Location | # sequences | |
| --- | --- | --- | --- |
|  |  | COI | Cytb |
| *Incertella incerta* | **Prairies** | | |
|  | Onefour | 9 | 10 |
|  | Cypress Hills AB | 6 | 6 |
|  | Cypress Hills SK | 4 | 4 |
|  | *Total* | *19* | *20* |
|  | **Peace River Region** | | |
|  | Dunvegan | 17 | 19 |
|  | **Yukon** | | |
|  | Robinson | 19 | 19 |
|  | Carmacks | 1 | 1 |
|  | *Total* | *20* | *20* |
| *Meromyza columbi* | **Prairies** | | |
|  | Onefour | 12 | 11 |
|  | Cypress Hills AB | 2 | 2 |
|  | Dinosaur | 4 | 4 |
|  | *Total* | *18* | *17* |
|  | **Peace River Region** | | |
|  | Dunvegan | 19 | 19 |
|  | Peace River | 2 | 2 |
|  | *Total* | *21* | *21* |
|  | **Yukon** | | |
|  | Carmacks | 5 | 5 |
|  | Takhini | 14 | 14 |
|  | *Total* | *19* | *19* |
| *Trixoscelis fumipennis* | **Prairies** | | |
|  | Aweme | 2 | 4 |
|  | Cypress Hills AB | 6 | 6 |
|  | Onefour | 5 | 5 |
|  | *Total* | 13 | 15 |
|  | Peace River Region | | |
|  | Dunvegan | 12 | 12 |
|  | Peace River | 9 | 9 |
|  | *Total* | 21 | 21 |
|  | **Yukon** | | |
|  | Robinson | 20 | 20 |
