# Supplementary material for: Phylogeography of higher Diptera in glacial and postglacial grasslands in western North America
Source: BMC Ecol. 2019 Dec 20;19:53. doi: 10.1186/s12898-019-0266-4 (PMC6923875; doi:10.1186/s12898-019-0266-4)
Supplement: Supplementary file 3 — Additional file 3. GenBank accession numbers for Diptera phylogeny. Additional taxa (with GenBank accession numbers) used for calculating divergence times. [file 12898_2019_266_MOESM3_ESM.docx]

**Additional file 3**: Additional taxa (with GenBank accession numbers) used for calculating divergence times

| Taxon represented | Family | Species | COI accession | Cytb accession |
| --- | --- | --- | --- | --- |
| Chloropidae | Chloropidae | *Thaumatomyia notata* | KC192976 | KC177599 |
| Acalyptratae | Carnidae | *Hemeromyia anthracina* | FJ025644 | FJ025740 |
| Acalyptratae | Sphaeroceridae | *Copromyza* sp. JHK-2012 | JX260391 | JX887702 |
| Acalyptratae | Sphaeroceridae | *Rachispoda* sp. JFG-2010 | HM062544 | HM062566 |
| Acalyptratae | Drosophilidae | *Drosophila* sp. JFG-2010 | HM062530 | HM062555 |
| Acalyptratae | Heleomyzidae | *Epistomyia* sp. JHK-2012 | JX260392 | JX887703 |
| Cyclorrhapha | Phoridae | *Conicera dauci* | HM062538 | HM062562 |
| Cyclorrhapha | Lonchopteridae | *Lonchoptera tristis* | HM062534 | HM062558 |
| Cyclorrhapha | Platypezidae | *Platypeza* sp. JFG-2010 | HM062540 | HM062563 |
| Cyclorrhapha | Syrphidae | *Toxomerus marginatus* | HM062546 | HM062568 |
| Schizophora | Muscidae | *Spilogona* sp. JFG-2010 | HM062536 | HM062560 |
